# Multimodal Signal Analysis for Pain Recognition in Physiotherapy Using Wavelet Scattering Transform

**DOI:** 10.3390/s21041311

**Published:** 2021-02-12

**Authors:** Aleksandra Badura, Aleksandra Masłowska, Andrzej Myśliwiec, Ewa Piętka

**Affiliations:** 1 Faculty of Biomedical Engineering, Silesian University of Technology, Roosevelta 40, 41-800 Zabrze, Poland; ewa.pietka@polsl.pl; 2 Institute of Physiotheraphy and Health Science, Academy of Physical Education in Katowice, Mikołowska 72a, 40-065 Katowice, Poland; a.maslowska@awf.katowice.pl (A.M.); a.mysliwiec@awf.katowice.pl (A.M.)

**Keywords:** pain assessment, pain monitoring, physiotherapy

## Abstract

Fascial therapy is an effective, yet painful, procedure. Information about pain level is essential for the physiotherapist to adjust the therapy course and avoid potential tissue damage. We have developed a method for automatic pain-related reaction assessment in physiotherapy due to the subjectivity of a self-report. Based on a multimodal data set, we determine the feature vector, including wavelet scattering transforms coefficients. The AdaBoost classification model distinguishes three levels of reaction (no-pain, moderate pain, and severe pain). Because patients vary in pain reactions and pain resistance, our survey assumes a subject-dependent protocol. The results reflect an individual perception of pain in patients. They also show that multiclass evaluation outperforms the binary recognition.

## 1. Introduction

Pain is a distressing experience that is associated with actual or potential tissue damage with sensory, emotional, cognitive, and social components [1]. These components indicate the subjectivity of pain. The same stimulus of a common source, duration, and location can cause various patient-related reactions. Thus, apart from assessment through numbers, such as pain intensity ratings or pain threshold levels, pain should be assessed in a personal context and meaning [2]. Moreover, Herr et al. [3] listed few populations of patients who may have difficulty with self-reporting pain, among others, young children, people with intellectual disabilities, critically ill or unconscious patients, and adults with advanced dementia. Whereas, in these cases, patients can self-report pain by other sounds, gestures, head positions, or eye blink, obtained information can be skewed, due to, e.g., inexperience of a clinician or uncertainty of the reaction. The challenges of pain assessment lead to the development of automatic pain monitoring systems.

Multiple researches was carried out on automatic pain monitoring systems [4,5,6,7,8,9,10,11,12,13,14,15,16,17]. They vary with the source of pain, collected data, and types of getting information about patients subjective pain rate. Many of them were based on publicly available data sets. The BioVid Heat Pain Database includes data from 90 participants, healthy adults. The experiment was based on a carefully planned set of four-level thermal stimulation, including the calibration process. The database contains the following biopotentials: electrodermal activity (EDA), electrocardiography (ECG), electromyography of corrugator, zygomaticus, and trapezius muscles (EMG), and electroencephalography (EEG). The video of subjects’ faces and depth maps were also acquired [4]. A system for continuous estimation of pain intensity in a person-independent scenario was presented in [5]. The authors highlighted that pain recognition of the BioVid Database is efficient without personalization. Biosignals and video features were both applied. Several surveys were given by Lopez-Martinez et al. [6,7], whereas some of them were based only on biopotentials features. A regression algorithm that was based on recurrent neural networks was presented in [8]. A set of various modalities was acquired for the X-ITE Pain Database. Apart from ECG, EMG, EDA, face, and body video it includes thermal video data and audio signal. Moreover, two sources of pain were used in the survey: heat and electrical stimuli. The stimulation variants were recognized (short/phasic vs. long/tonic stimulation) in [15]. The efficiency of fusion of various modality combinations was also tested. Because the database is relatively new (2019), the research is still ongoing. Data from 129 subjects with shoulder pain were collected for the UNBC-McMaster Shoulder Pain Database [9]. The study protocol assumed eight motion tests. The active tests were performed with the patient in a standing position, while passive tests were performed by a physiotherapist who moved the limb. The pain was assessed by a self-report after examination and by the offline independent rating based on the video of a scene. Video of the face was collected as well. In [10], the authors proposed the method of pain intensity regression using a convolutional neural network (CNN). The spatio-temporal information of face video was used in [11]. The authors compared the performance of the descriptors on both the BioVid and UNBC-McMaster databases. The EmoPain Dataset [12] stands for a similar one to the UNBC Database. It includes data from 22 patients with chronic lower back pain performing physical exercises. Video, audio signal, and EMG of trapezius, lumbar, and paraspinal muscles were acquired. The research on this database focus on motion capture fused with EMG analysis [13,14]. Although there are few more pain databases [16,17], we do not describe them here due to indirect relation to our study.

Research on UNBC-McMaster and EmoPain seems to respond to the need of pain monitoring in physiotherapy. Face activity descriptors we determined to detect and estimate the pain intensity in patients suffering from shoulder pain during motion tests [18]. In a two-class problem, the authors report the accuracy at 92%. In [14], healthy participants and patients with chronic musculoskeletal pain were examined. The study protocol included *sit-to-stand* and *full trank flexion* physical exercises. The authors achieved the accuracy at about 70–95% in three-class pain level recognition using kinematic and EMG features. It was observed that patients suffering from chronic pain adjust the strategy of performing exercises (e.g., using alternative parts of the body) due to the fear of pain [12]. Thus, body movements reflected protective pain-related behaviors. The emotional state (like fear of pain, anxiety, or demotivation) is highly connected with the efficacy of therapy that is based on self-exercises [12,13]. Some factors make physical exercises incomparable to manual therapy. In physical exercises, the patient himself doses parameters that may affect the severity of painful feeling. Fascial therapy is a direct intervention in the patients tissue, and it largely depends on the physiotherapist’s experience and intuition. Protective behaviors do not occur because the pain source is external and cannot be controlled by the patient. Moreover, it was observed that, during this specific procedure, pain appears mainly unexpectedly. It makes the mentioned pain level assessment solutions not applicable to the fascial therapy. It is one of the methods used in the treatment of pain syndromes of the musculoskeletal system caused by overload (mainly due to the need to hold the position for a long time [19]). Its goal is to reduce the tension and improve the flexibility of the muscle tissue. These aims are obtained by point pressure or deep rubbing combined with a slow movement along the muscle fibers. It results in improving blood circulation and, thus, increases tissue oxygenation [20]. The therapy course can be defined as an individually selected set of variable stimuli (in terms of strength and plane) transmitted through the physiotherapists hand. During its performance, it is necessary to warm-up tissues from superficial to deeper ones [21,22]. This effect is achieved by gradually increasing the strength and intensity of the stimulus [23]. The force of the procedure must not exceed the range that may possibly harm the patient. In a therapy, which is not assisted by a pain monitoring system, nociceptive pain information is the only measure that can be a real-time feedback on the intensity of the therapy [24], which is connected withrapid behavioral reactions, such as grimacing, bracing, and sighing [25]. Because pain is a subjective experience, the therapy of pain-resistant patients may result in tissue damage, while sensitive patient reactions could limit the therapy outcomes. Therefore, using an automatic pain assessment system could aid the therapy to be effective but harmless [26]. Additionally, such a system could help in avoiding many requests regarding the perceived pain intensity, which could be annoying for the patient. To the best of our knowledge, no research on pain-related reaction monitoring during manual physiotherapy has been carried out so far.

The goal of this study is to develop a pain-related reaction (PRR) assessment method that is applied to fascial therapy. A designed and implemented multimodal setup acquires and synchronizes patient data during physiotherapy. An advanced signal analysis approach followed by feature extraction in a time and frequency domain yields a vector that is subjected to a classification procedure that enables the detection of changes in the PRR level that may not be visible in every registered signal. The appearance of the pain-dependent changes in various signals differs between patients. Thus, a patient-related approach has been developed to distinguish the *no-pain*, *moderate pain*, or *severe pain* condition.

The paper is organized, as follows: the next section describes the collected dataset and methods, i.e., feature extraction from the synchronized, multimodal data, and classification approaches that are employed for the PRR assessment. Section 3 presents the outcomes of the study. Finally, we discuss the results considering the system’s intended use for fascial therapy and compare it to research outcomes published so far. We also present plans for further work.

## 2. Materials and Methods

For this study, we collected data from 34 subjects (23 female, 11 male, mean age 44 ± 15) undergoing fascial manual therapy. Many of them were regular patients of the physiotherapy clinic. Data for one more patient were registered, but excluded from the survey. The patient was not able to express the pain level clearly. The study was taken in an isolated, quiet lab. The therapy focused on neck and arm muscles. First, a 30-second data segment with no pain induction was acquired. Subsequently, the 2–3 min. therapy started and entire data set was recorded. Patients were in a sitting position. Information about pain feelings was expressed by saying a number in the range of 0–10 (where 0 stands for no-pain and 10 for maximum pain). One physiotherapist carried out the therapy. The video of the scene was recorded for offline analysis. All of the subjects were informed about the survey procedure and signed the agreement.

**Figure 1:**
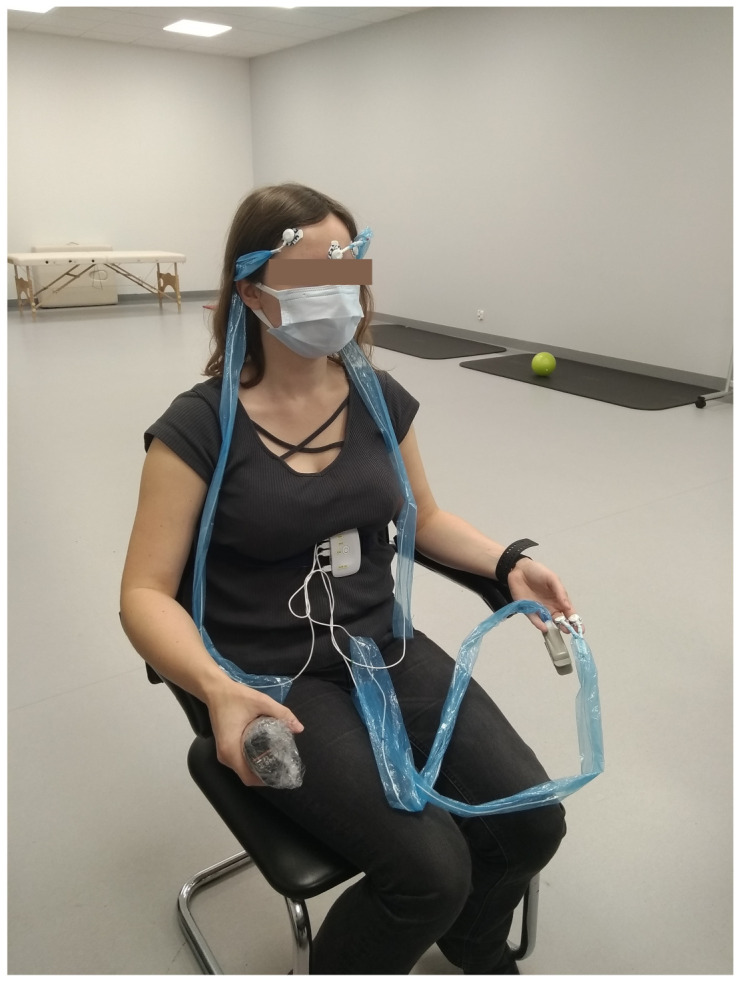
Device set.

Signals, including electrodermal activity (EDA), electromyography (EMG), respiratory (RSP), blood volume pulse (BVP), and hand force (GRIP), were registered by wearable devices: RespiBan, Empatica, and K-Force (Figure 1). EDA sensors were attached to the middle part of the index and middle fingers. EMG electrodes on the forehead registered signals from the corrugator superciliimuscle. The RSP signal was collected from a device that was placed on a chest stripe. The K-Force dynamometer was held in the right hand and it might be subconsciously squeezed, depending on the subjective pain intensity. The data were registered by the PainMonit platform, which delivers synchronized time-series with a pain label [27].

### 2.1. Signal Preprocessing

Several steps are performed at the processing stage. First, the signals are resampled with anti-aliasing FIR low pass filters to their desired sampling frequencies (EMG sampled with 256 Hz, BVP 64 Hz, EDA 8 Hz, RSP 8 Hz, and GRIP 75 Hz). Afterwards, the Gaussian-weighted moving average filter is applied to the EDA signal. Next, a function that detects a rapid change is required due to the sudden occurrence of the pain. It is based on Greco et al. [28] convex optimization approach. The model describes the skin conductance as a sum of three components: the tonic component, the phasic component, and an additive white Gaussian noise component that incorporates model prediction errors, measurement errors, and artifacts. The tonic component is responsible for the baseline’s slow drifts, while the phasic one reflects the stimulus’s rapid reactions. During the physiotherapy, a sudden pain results in a sweat glands response, thus the phasic component is employed for further analysis. It is modeled by the Bateman function, and followed by the Laplace transform. The algorithm is described in more detail in [27].

### 2.2. Feature Extraction

A deep scattering spectrum [29] is used at this phase. The algorithm computes a cascade of wavelet decompositions and modulus operators. Invariant to the time shift, it outperforms classically computed spectrograms. Mel-frequency cepstral coefficients overcome this problem by averaging over bands in the frequency domain, but, on the other hand, they lose information and can be used up to 25 ms time intervals. Scattering transform can preserve energy even for larger time scales. These factors seem to be crucial, especially in the low-frequency RSP signal, which is sensitive to time-warping deformations [30]. The deep scattering algorithm relies on computing scattering and scalogram coefficients throughout the following layers using banks of wavelet filters. First-order scattering coefficients are obtained by averaging the wavelet modulus coefficients with ϕ:

(1) $$S_{1}x\left(t,\lambda_{1}\right)=|x*\psi_{\lambda_{1}}|*\phi \left(t\right),$$

where $\psi_{\lambda_{1}}$ stands for the first wavelet filter bank and *x* is the original signal. Next, layer of the transform is received by applying the new wavelet modulus coefficients:

(2) $$\begin{array}{cc} U_{2}x\left(t,\lambda_{2}\right) & =||x*\psi_{\lambda_{1}}|*\psi_{\lambda_{2}}|,\\ S_{2}x\left(t,\lambda_{2}\right) & =||x*\psi_{\lambda_{1}}|*\psi_{\lambda_{2}}|*\phi \left(t\right). \end{array}$$

**Figure 2:**
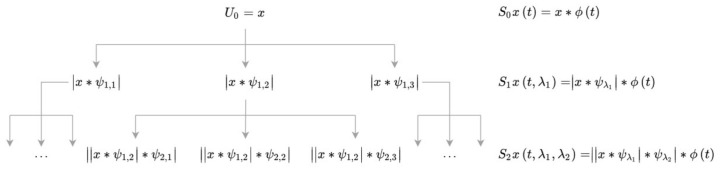
Wavelet scattering spectrum transform.

**Figure 3:**
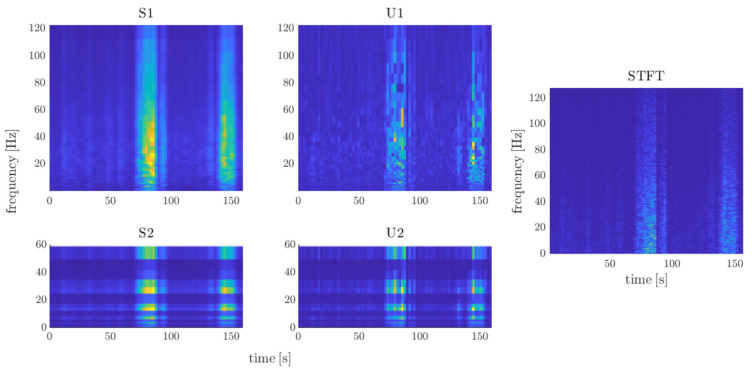
Output of the scattering spectrum transform ($S_{1}$, $U_{1}$, $S_{2}$, $U_{2}$) and short-time Fourier transform for electromyography (EMG) signal registered during a painful therapy. All of the values are normalized to the 0–1 range. Various energy distribution between transforms can be observed in subbands. Because scattering transform assumes a decreasing number of voices per octave, the time resolution increases in the following layers (see separable frames in $U_{2}$ at high energy around 30 Hz subband).

Figure 2 presents the process of determining the next coefficients of the transform. While scattering coefficients ($S_{n}$) imply invariance to translations, they remove high frequencies from the signal. These are recovered by the wavelet modulus transform ($U_{n+1}$). Wavelet filters used for the next transform layers differ with octave resolutions. For high-frequency signals (EMG, BVP), we apply Morlet wavelets [31] with eight, four, and one voice per octave (three-layer transform). For low-frequency signals (EDA, RSP), eight and one voices are used. Figure 3 shows the output of the transform as compared to the classical spectrogram.

The outputs of the next transform layers differ in the number of coefficients in the time domain due to the information compression. Outputs (scattergrams $S_{n}$ and scalograms $U_{n}$ for n>1) are interpolated with fast Fourier transform (FFT) to obtain an equal number of coefficients. Finally, we determine the energy for every single frame of scalogram and scattergram. Thus, 20 features are extracted using wavelet scattering transform.

For EMG and RSP, the spectral entropy is determined:

(3) $$\begin{array}{c} H\left(t\right)=-\sum_{m=1}^{N}P\left(t,m\right)\log_{2}P\left(t,m\right), \end{array}$$

where $P\left(t,m\right)$ stands for probability distribution:

(4) $$P\left(t,m\right)=\frac{S\left(t,m\right)}{\sum_{f}S\left(t,f\right)}$$

and $S\left(t,m\right)$ is a power spectrum. A power spectrum window width relates to the wavelet transform window width (4 s, 50% overlap).

Apart from wavelet and spectral features, maximum value, median, standard deviation, and absolute amplitude of the signal are determined in each window. Furthermore, the 1st derivative of the signal is subjected to the analysis. The maximum, mean value, and standard deviation are found. Finally, the feature vector consists of 33 elements. Subject-based normalization of the data while using z-score standardization is carried out.

### 2.3. Pain Labeling

**Figure 4:**
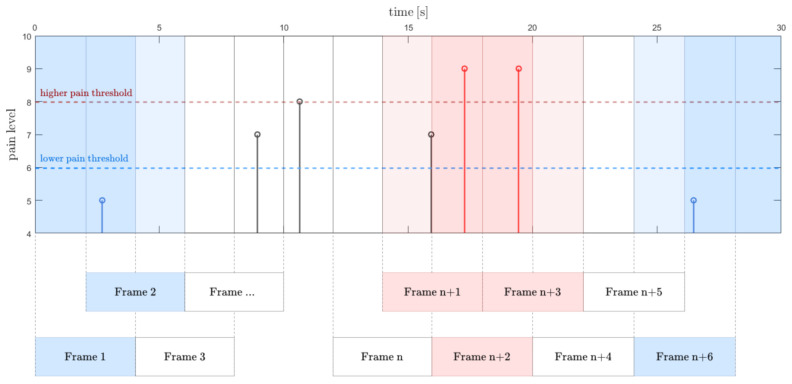
Frame labeling rule. *No-pain*, *moderate pain*, and *severe pain* intervals are indicated by two pain thresholds. If several values occurred, the highest is taken into account.

Based on patient rates, we obtain discrete pain level values over the time. A backward time shift is applied due to the possible delayed reaction of the patient. Subsequently, the pain labels vector is divided into frames of a width corresponding to the feature vector frames. A single frame is tagged with the maximum pain level value that occurred within this frame. Because the pain resistance is highly subject-dependent, an approach with double pain threshold is applied (Figure 4). The label below the lower threshold is assumed to address no or a very low pain sensation. The higher threshold indicates the borderline of a severe pain. The data between the two thresholds are assigned to the third group. Thus, each frame is labeled as: *no-pain*, *moderate pain*, or *severe pain* frame (Figure 4).

### 2.4. Classification

Two classification approaches are tested. The support vector machine (SVM) stands for the first one. The SVM [32] finds the hyperplane that separates observations into two classes with a maximum margin. It provides set of adjustment properties. One of them is a kernel function, which transforms data to a higher dimension for better separation [33]. Here, the Gaussian kernel function is used.

The adaptive boosting algorithm (AdaBoost) is used for the second classification attempt. It assumes the collection of weak learners (here, decision trees are used). The training error drops exponentially because a single tree performs slightly better than random guessing. The algorithm sequentially modifies the weights of the observations. The misclassified ones are boosted. Hence, the model is focused on the problematic examples in the training set [34]. The depth of the single tree has an influence on predictor importance in a training process. Here, the branch node per tree was set to 20.

**Figure 5:**
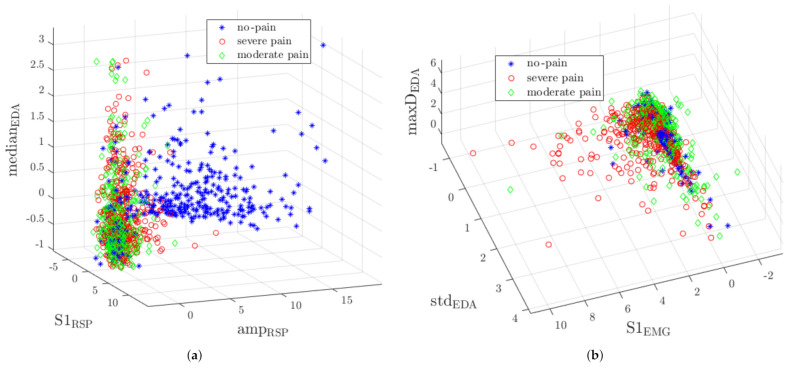
The distributions of representative cases in three-dimensional feature space: (**a**) presents well separable *no-pain* observations (amplitude of respiratory (RSP), the *S*_1_ energy of RSP and median of electrodermal activity (EDA) distribution), (**b**) reflects partial overlaping of severe and moderate pain classes (*S*_1_ energy of EMG, standard deviation of EDA, and maximum of EDA 1st derivative distribution).

Figure 5 presents two samples of three-dimensional feature distribution. One can notice that *no-pain* observations are mostly detached from other classes yet still some samples overlap (Figure 5a). Because *severe pain* and *moderate pain* classes overlap, the classification is not a trivial task. An EMG signal features the highest discriminant power, as shown in Figure 5b.

## 3. Results

**Figure 6:**
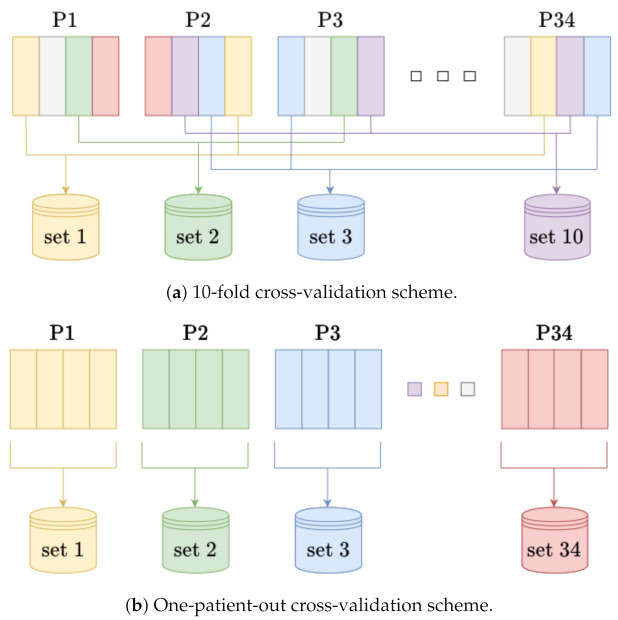
Illustration of cross-validation schemes. In (**a**) frames for test and train sets are selected randomly, whereas data from a single patient could be included in both. Otherwise, for (**b**) data from a single patient stand for the test set and the others for the training set. In both (**a**) and (**b**) the numerical balance throughout the classes is maintained.

Four experiments were carried out. We performed three binary classifications (*severe pain* vs. *no-pain*, *severe pain* vs. *moderate pain*, *moderate pain* vs. *no-pain*). In the first experiment, the training and test sets included randomly selected frames. A 10-fold cross-validation was used for testing. The assessment involved five metrics: accuracy, sensitivity, precision, specificity, and F1 score. Table 1 presents the results. The second experiment was based on the one-patient-out rule (Table 2). Two other experiments evaluate the multiclass approach. The data were assigned to one of three classes: *no-pain*, *moderate pain*, and *severe pain*. In the multiclass approach, metrics, such as accuracy, sensitivity, etc., were determined separately for each class. Results of 10-fold cross-validation and one-patient-out cross-validation are given in Table 3 and Table 4, respectively. Because SVM multiclass performance was mostly worse than 0.50, the results are not presented here. Figure 6 presents the details of experiments validation.

All four experiments were run ten times with a newly selected random data set. The presented results relate to the shift of pain markers by 2 s backward.

For comparison, in addition to the wavelet scattering features analysis, a spectrogram frames energy (calculated with the short-time Fourier transform, STFT) approach has been tested. It reduces the number of features to just one parameter for a single modality instead of four or six scattering features. In this case, the entire feature vector consisted of 17 elements. Table 5 and Table 6 present the results.

## 4. Discussion

**Figure 7:**
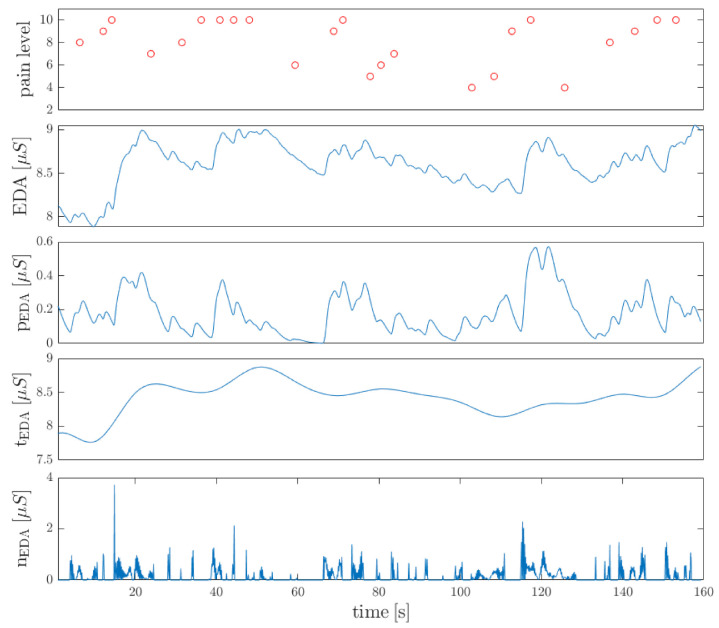
The results of convex optimization algorithm applied to resampled EDA signal: phasic ($p_{EDA}$), tonic ($t_{EDA}$), and noise ($n_{EDA}$) components. Significant increase in $p_{EDA}$ amplitude can be noticed especially for pain labels greater than 8 (top chart). The data come from the therapy session.

We obtained efficiency at different levels, depending on the experiment and classes to be separated. For binary classification, the best results were observed for *severe pain* vs. *no-pain* recognition (cross-validation F1 score at 0.95). *moderate pain* vs. *no-pain* classification yielded similar results in both validation approaches (0.87). Relatively poor outcomes were observed for *severe pain* vs. *moderate pain*. On one hand, the subjective pain thresholds could cause incorrect pain label assignment swinging between the two levels. On the other hand, the maximum pain level was not invoked by the physiotherapist (therapy was not intended to torment the patient), so the threshold could not even be exceeded for pain-resistance patients. It also explains the results of *moderate pain* classification in the multiclass approach (F1 score at 0.69/0.63). Indeed, even based on patients behavior during the study, it was difficult to distinguish between the two conditions. Pain level, which was recognized by a patient as a severe one at the beginning of the therapy, was diagnosed as mild with continued therapy. Moreover, measuring a low-intensity pain (which gives low amplitude responses) is the most challenging goal to be achived in automatic pain severity assessment systems [35]. Binary classification yielded high sensitivity when *no-pain* standed for the negative class. Indeed, the EDA signal seems to reflect this phenomenon most reliably, since the low-level plateau is disturbed, even when mild pain occurs. The phasic component of convex optimization algorithm applied to EDA signal enhances rapid changes. The EDA decomposition with corresponding pain labels is shown in Figure 7. Whereas sensitivity stands for essential metrics in the severe pain recognition, the multiclass approach provided quantitatively better results than binary classification (sensitivity at 0.82 and 0.64 for *severe pain* and *moderate pain* classes, respectively, whereas binary classification at 0.47 for *severe pain* vs. *moderate pain* in one-patient-out cross-validation sounds unacceptable). Multiclass approach reflects the physiotherapy requirements, where certain PRR levels are distinguished, rather than the only a pain onset.

For binary classification, AdaBoost performed better than SVM. Although the AdaBoost algorithm was already used for pain assessment with the UNBC-McMaster database, camera/facial expressions only stood for modality [36,37]. There are several works where SVM was applied to the BioVid database. In [38], the authors achieved the accuracy at 0.53–0.77 in binary classification between five-level pain intensities (one-patient-out cross-validation). The F1 score around 0.80 was obtained for binary no-pain and maximum pain [4], whereas head-pose features were also used. In [39], only biomedical signals were used for random forest classification with accuracy at 0.85 (no-pain vs. maximum pain). Therefore, this survey is the first one where the AdaBoost has been applied to biomedical signals and seems to respond well to challenges of PRR assessment problem. Because SVM uses the entire feature set with equal weights to build the model, AdaBoost finds and enhances borderline observations. Feature selection was not conducted for a reliable comparison of classifiers. However, AdaBoosts advantage over SVM shows that decision trees, used as weak learners, provide a satisfactory build-in feature selection. The results also show that the approach with a feature set including wavelet scattering features in almost all cases outperforms the model based on the STFT feature set. Wavelet scattering transform provides accurate measurements of frequency intervals between harmonics while preserving time resolution. It is also insensitive to time-warping. Moreover, the next layers of the transform have reduced representation, leading to faster implementation [29].

Because lots of research on automatic pain recognition was carried out, to the best of the authors knowledge no attempts have been made for PRR monitoring in fascial therapy. While research on, e.g., the BioVid database, considers detailed study protocol, including a growth rate and duration of the temperature stimuli, the fascial therapy is a dynamic procedure. The therapist adjusts force, pressure duration, and pressure plane on a specific point on the neck/arm, depending on the tissue and patient feedback. Hence, the patient reaction is unpredictable in advance. What is more, it is difficult to assess how much energy was transferred to the patient or even to compare the course of one therapy to another. That all makes planning similar experiments among patients impossible [40,41]. The lack of pain reference given to patients was the challenging task in this survey (which was given e.g., in [15,38]). A physiotherapist cannot begin the therapy inducing a high-level pain due to the tissue warming-up requirement. Therefore, subjects could rate the pain with a certain bias. Because the interpretation of the pain labels as the Numeric Rating Scale (NRS) or Visual Analogue Scale (VAS) [42] in our study is pointless, we considered them rather as a set of increasing and decreasing values. Although low-frequency signals were analyzed, our algorithm uses relatively short time frames. Pain intensity can change rapidly during therapy: it was observed that a lack of pain could occur just after the severe pain. Moreover, an online approach will be considered in further development, thus a short-time analysis is required.

Individual reactions were observed among patients. Some of them showed almost no facial expressions, while others did a lot. Although some single modalities seem to follow the PRR level well, they may not be always reliable. For example, noticeable changes were observed in EDA with the onset of pain, yet the signal also reflects reactions to emotions [35]. Thus, the multimodal dataset and usage of feature fusion seem to be crucial in PRR assessment. Apart from that, it is worth noting that one-patient-out performed worse than 10-fold cross-validation in most of the experiments. It confirms the lack of versatile reaction to pain during physiotherapy. In consequence, a subject-dependent approach to this problem is desired. Hence, in our further work, we plan to develop the patient-specific model. The personalized model will likely improve the efficiency and enable taking a closer look at the subjective patient reaction. Additionally, an attempt to recognize more than three classes may perform better and more precisely reflect the nature of pain in manual physiotherapy.

## Figures and Tables

**Table 1:** Binary classification results of 10-fold cross-validation.

	Severe Pain vs. No-Pain		Severe Pain vs. Moderate Pain		Moderate Pain vs. No-Pain	
	AdaBoost	SVM	AdaBoost	SVM	AdaBoost	SVM
Accuracy	0.94 ± 0.02	0.63 ± 0.10	0.79 ± 0.04	0.54 ± 0.10	0.85 ± 0.04	0.61 ± 0.16
Sensitivity	0.97 ± 0.02	0.64 ± 0.35	0.79 ± 0.07	0.76 ± 0.25	0.95 ± 0.04	0.39 ± 0.40
Precision	0.92 ± 0.04	0.66 ± 0.08	0.79 ± 0.05	0.59 ± 0.15	0.80 ± 0.05	0.73 ± 0.05
Specificity	0.91 ± 0.04	0.67 ± 0.20	0.79 ± 0.06	0.37 ± 0.39	0.76 ± 0.08	0.88 ± 0.13
F1 score	0.95 ± 0.02	0.58 ± 0.25	0.79 ± 0.05	0.61 ± 0.05	0.87 ± 0.04	0.38 ± 0.38

**Table 2:** Binary classification results of one-patient-out cross-validation.

	Severe Pain vs. No-Pain		Severe Pain vs. Moderate Pain		Moderate Pain vs. No-Pain	
	AdaBoost	SVM	AdaBoost	SVM	AdaBoost	SVM
Accuracy	0.82 ± 0.15	0.56 ± 0.18	0.64 ± 0.10	0.50 ± 0.00	0.85 ± 0.12	0.62 ± 0.17
Sensitivity	0.77 ± 0.32	0.40 ± 0.49	0.47 ± 0.27	0.59 ± 0.49	0.95 ± 0.07	0.37 ± 0.48
Precision	0.89 ± 0.13	0.39 ± 0.35	0.74 ± 0.16	0.50 ± 0.00	0.81 ± 0.14	0.76 ± 0.13
Specificity	0.88 ± 0.16	0.71 ± 0.30	0.80 ± 0.19	0.40 ± 0.49	0.74 ± 0.24	0.87 ± 0.23
F1 score	0.77 ± 0.28	0.32 ± 0.40	0.52 ± 0.24	0.39 ± 0.33	0.87 ± 0.09	0.31 ± 0.42

**Table 3:** Multiclass evaluation results of 10-fold cross-validation (AdaBoost).

	Severe Pain	Moderate Pain	No-Pain
Accuracy	0.84 ± 0.03	0.77 ± 0.04	0.91 ± 0.02
Sensitivity	0.77 ± 0.07	0.77 ± 0.07	0.75 ± 0.07
Precision	0.76 ± 0.06	0.64 ± 0.05	0.98 ± 0.03
Specificity	0.87 ± 0.04	0.78 ± 0.05	0.99 ± 0.01
F1 score	0.76 ± 0.05	0.69 ± 0.05	0.85 ± 0.05

**Table 4:** Multiclass evaluation results of one-patient-out cross-validation (AdaBoost).

	Severe Pain	Moderate Pain	No-Pain
Accuracy	0.79 ± 0.17	0.80 ± 0.10	0.92 ± 0.13
Sensitivity	0.82 ± 0.25	0.64 ± 0.30	0.81 ± 0.21
Precision	0.70 ± 0.21	0.78 ± 0.16	0.96 ± 0.18
Specificity	0.79 ± 0.20	0.88 ± 0.14	0.97 ± 0.13
F1 score	0.73 ± 0.21	0.63 ± 0.26	0.87 ± 0.19

**Table 5:** Multiclass evaluation results of 10-fold cross-validation (AdaBoost): comparison of short-time Fourier transform (STFT) features and wavelet scattering features approaches.

	Severe Pain		Moderate Pain		No-Pain	
	scattering	STFT	scattering	STFT	scattering	STFT
Accuracy	0.84 ± 0.03	0.80 ± 0.03	0.77 ± 0.04	0.73 ± 0.04	0.91 ± 0.02	0.91 ± 0.02
Sensitivity	0.77 ± 0.07	0.63 ± 0.09	0.77 ± 0.07	0.79 ± 0.08	0.75 ± 0.07	0.74 ± 0.06
Precision	0.76 ± 0.06	0.74 ± 0.06	0.64 ± 0.05	0.57 ± 0.05	0.98 ± 0.03	0.98 ± 0.03
Specificity	0.87 ± 0.04	0.89 ± 0.04	0.78 ± 0.05	0.70 ± 0.06	0.99 ± 0.01	0.99 ± 0.01
F1 score	0.76 ± 0.05	0.67 ± 0.06	0.69 ± 0.05	0.66 ± 0.05	0.85 ± 0.05	0.84 ± 0.04

**Table 6:** Multiclass evaluation results of one-patient-out cross-validation (AdaBoost): comparison of STFT features and wavelet scattering features approaches.

	Severe Pain		Moderate Pain		No-Pain	
	scattering	STFT	scattering	STFT	scattering	STFT
Accuracy	0.79 ± 0.17	0.77 ± 0.14	0.80 ± 0.10	0.77 ± 0.09	0.92 ± 0.13	0.91 ± 0.14
Sensitivity	0.82 ± 0.25	0.76 ± 0.30	0.64 ± 0.30	0.57 ± 0.34	0.81 ± 0.21	0.84 ± 0.17
Precision	0.70 ± 0.21	0.68 ± 0.20	0.78 ± 0.16	0.75 ± 0.16	0.96 ± 0.18	0.95 ± 0.17
Specificity	0.79 ± 0.20	0.77 ± 0.21	0.88 ± 0.14	0.87 ± 0.15	0.97 ± 0.13	0.95 ± 0.19
F1 score	0.73 ± 0.21	0.66 ± 0.22	0.63 ± 0.26	0.56 ± 0.29	0.87 ± 0.19	0.88 ± 0.16

## Data Availability

Data sharing is not applicable to this article.
